# Using Implicit-Solvent Potentials to Extract Water Contributions to Enthalpy–Entropy Compensation in Biomolecular Associations

**DOI:** 10.1021/acs.jpcb.3c03799

**Published:** 2023-07-26

**Authors:** Shensheng Chen, Zhen-Gang Wang

**Affiliations:** Division of Chemistry and Chemical Engineering, 1200 E California Blvd, California Institute of Technology, Pasadena, California 91125, United States

## Abstract

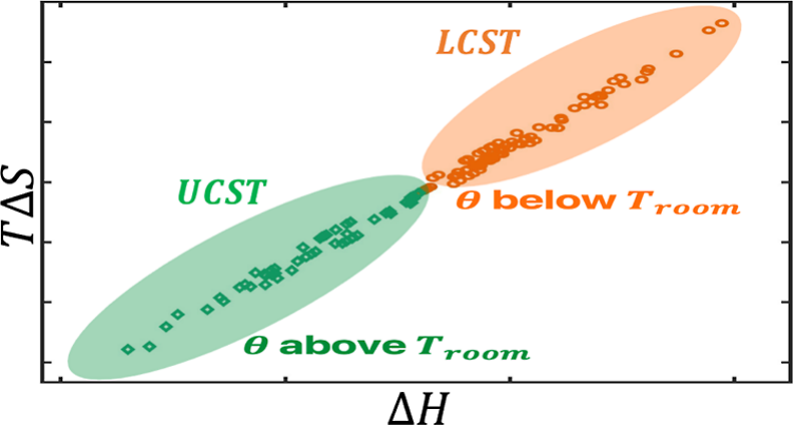

Biomolecular assembly typically exhibits enthalpy–entropy compensation (EEC) behavior whose molecular origin remains a long-standing puzzle. While water restructuring is believed to play an important role in EEC, its contribution to the entropy and enthalpy changes, and how these changes relate to EEC, remains poorly understood. Here, we show that water reorganization entropy/enthalpy can be obtained by exploiting the temperature dependence in effective, implicit-solvent potentials. We find that the different temperature dependencies in the hydrophobic interaction, rooted in water reorganization, result in substantial variations in the entropy/enthalpy change, which are responsible for EEC. For lower-critical-solution-temperature association, water reorganization entropy dominates the free-energy change at the expense of enthalpy; for upper-critical-solution-temperature association, water reorganization enthalpy drives the process at the cost of entropy. Other effects, such as electrostatic interaction and conformation change of the macromolecules, contribute much less to the variations in entropy/enthalpy.

## Introduction

The association between biomolecules such as proteins and nucleic acids in an aqueous environment is the molecular basis of life. The specific interaction between biomolecules, or molecular recognition, guides cellular organization and functions. Specific interactions in ligand–protein binding is also the foundation of pharmaceuticals. Despite decades of research in the study of biomolecular recognition, a molecular understanding of the driving force for their association behavior remains unclear. A long-standing puzzle in biomolecular association is the molecular origin of the enthalpy–entropy compensation (EEC).^1−4^ EEC refers to the contributions to the thermodynamic driving force in the binding between two biomolecules: with small modifications in the chemical structure of the binding species, while the change in Gibbs energy (Δ*G* = Δ*H* – *T*Δ*S*) of binding remains relatively small, there are substantial variations in enthalpy (Δ*H*) and entropy (Δ*S*), and the changes in the two contributions effectively compensate each other in the free-energy contribution. Such a compensating behavior seems to hold in the association between wide classes of biomolecules.^4−13^ While the magnitude of the Gibbs free-energy change is most probably a consequence of natural evolution^14−17^—biological interactions cannot be too strong or too weak—both the wide range of the entropy (and enthalpy) variation and the origin of the entropy change remain poorly understood. An early explanation of the compensation effect invoked the intuitive picture where binding results from the favorable energetic attraction at the cost of conformation entropy loss.^18,19^ However, such an argument does not explain the prevalence of entropy-driven association systems and the large entropy changes in EEC cases where no significant conformation changes are involved.^4,20,21^

Water, being the ubiquitous solvent for biomolecular association, is widely believed to play a key role in EEC.^1,2,21−23^ Water reorganization, such as local structural changes in the hydrogen bonding network and water release from or adsorption to the biomolecules, is often invoked as being responsible for the driving forces in the biomolecular associations. It has been generally recognized that water reorganization plays a pivotal role in the organization of living matter,^24^ including protein folding,^25−28^ formation of bio condensates,^29^ and formation of membranes/micelles.^30,31^ In several experimental reports of EEC, water reorganization has been suggested to be the major contributor to the entropy/enthalpy change.^32−34^ In a convincing study, Breiten et al. examined the association between a crystallized protein and a series of rigid ligands in water;^20^ since the binding involves no obvious changes in the molecular conformation, they concluded that water reorganization is the only explanation for the observed EEC behavior.^20,21^ However, the extent to which water reorganization contributes to the entropy and enthalpy changes in biomolecular associations and the connection between these changes and the observed EEC behavior remain poorly understood.

Calculation of the free-energy contributions from water reorganization in macromolecular association by quantum, first-principles methods is currently an impossible task. However, in our recent work, we showed that water reorganization entropy/enthalpy in an electrostatically driven association can be obtained by exploiting the temperature dependence of the water-mediated electrostatic interaction.^35^ For polyelectrolyte complex coacervation, we found that water reorganization entropy, rather than the commonly believed counterion release entropy, is the primary entropy-driving force for many of the experimentally relevant polyelectrolyte systems. Thus, a promising approach to understanding the water reorganization entropy/enthalpy effects on biomolecular association is by analyzing appropriately constructed temperature-dependent, effective water-mediated interaction potentials relevant to biomolecular systems. Such temperature-dependent, coarse-grained potentials have been developed by Dignon et al.^36^ to successfully predict the liquid–liquid phase separation of disordered proteins.

In this article, we present a phenomenological approach to understanding EEC, in which the water reorganization entropy and enthalpy are captured by temperature-dependent, coarse-grained potentials. We illustrate our approach by studying the association between two model oppositely charged polymers using molecular dynamics simulation. From the analysis of the potential of mean force (PMF), we find that the entropy and enthalpy change from water reorganization are the major contributions to the free-energy change in the binding process and that these two components of the driving force compensate for each other. The substantial variations in the entropy and enthalpy changes observed in systems exhibiting EEC behavior arise from the different temperature dependence of the hydrophobic/hydrophilic interactions. For association in lower-critical-solution-temperature (LCST) systems, water reorganization entropy dominates the favorable free-energy change, compensated by unfavorable enthalpy. For association in upper-critical-solution-temperature (UCST) systems, water reorganization enthalpy provides the favorable free-energy driving force, compensated by unfavorable entropy. The magnitude of entropy/enthalpy change from water reorganization depends strongly on the difference between the operational temperature *T* (room temperature in this study) and the (mean-field) θ temperature of the polymers.

## Methods

### Water Reorganization Entropy/Enthalpy from Implicit-Solvent Potentials

The key to extracting water reorganization entropy/enthalpy from implicit-solvent, coarse-grained models is the recognition that the interaction potentials are PMFs. As such, the PMF is the interaction free energy that contains both entropic and energetic contributions,^37^ with the water degrees of freedom reflected in the temperature dependence of the PMFs. (Strictly speaking, the free-energy change refers to a Helmholtz free-energy change, but for liquid systems under normal conditions, it is approximately the Gibbs free-energy change. For this reason, we shall refer to the energy change as the enthalpy change). The PMF between a pair of solutes in water *w*(*r*) can be generally written as *w*(*r*) = *u*(*r*) + Δ*w*(*r*, *T*), where *u*(*r*) is the direct interaction potential in the absence of water and Δ*w*(*r*, *T*) is the water-mediated contribution.^38^ Note that Δ*w*(*r*, *T*) is temperature-dependent, reflecting the effects of integrating the solvent degrees of freedom. Knowing the exact form of *w*(*r*, *T*), we can calculate the change in entropy and enthalpy in molecular association as^37^
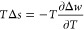
1and
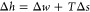
2where Δ refers to the change before and after solute association. The temperature dependence in *w* is due exclusively to the water-mediated contribution, Δ*w*(*r*, *T*). Therefore, the calculated *T*Δ*s* from eq 1 is the water reorganization entropy. Consequently, Δ*h* calculated by eq 2 contains the enthalpy from solvent reorganization. From eqs 1 and 2, a strong temperature dependence in *w* will result in significant contributions to the free-energy change from water reorganization. The key to extracting the water reorganization entropy and energy in biomacromolecular association, therefore, is knowing the temperature dependence in the relevant effective potentials.

Biomolecular association in water is generally considered to consist of two types of effective interactions: ionic electrostatic interactions and non-ionic interactions. Following the common usage,^39−41^ we use the term hydrophobic interaction to refer to all non-ionic interactions. Both electrostatic and hydrophobic interactions in water have strong temperature dependence.^42−44^ Below, we discuss the temperature dependence in these two types of interactions and how it relates to the water reorganization entropy/enthalpy. We then use coarse-grained molecular dynamics simulation to study the association between two generic oppositely charged polymers, with the aim of exploring the role of water reorganization in EEC.

### Water Reorganization in Electrostatic Interactions

**Figure 1:**
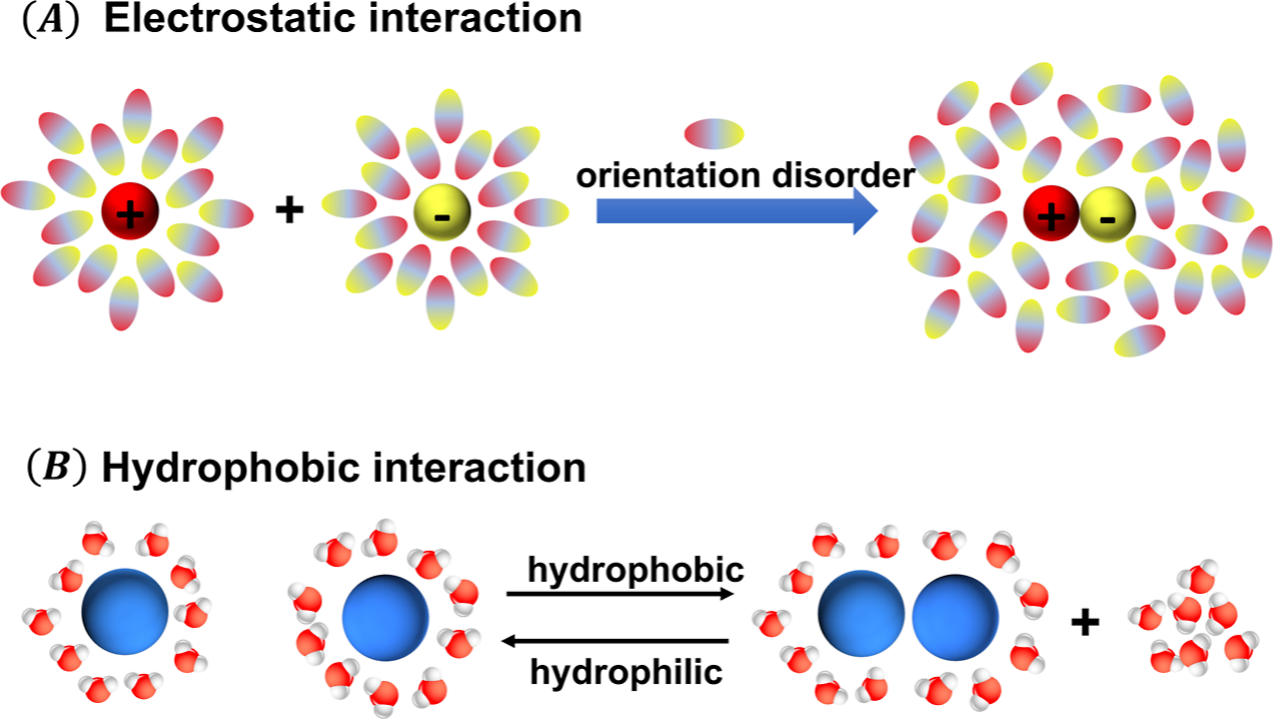
Schematics showing how water reorganization mediates effective interactions. (A) Electrostatic interactions are mediated by the orientation of water dipoles. (B) Hydrophobic interactions are mediated by local water restructuring and/or release/adsorption of water molecules.

At the coarse-grained level, the effective interaction between two ions in water is described by the Coulomb potential
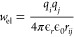
3where *r*_*ij*_ is the distance between two charges *q*_*i*_ and *q*_*j*_, ϵ_*r*_ is the dielectric constant of the solvent, and ϵ_0_ is the vacuum permittivity. In this treatment, all solvent degrees of freedom are subsumed into the dielectric constant ϵ_*r*_. The dielectric constant of water has a strong temperature dependence,^42^ pointing to the significant role played by entropy in the electrostatic interaction due to water reorganization.^45,46^ In our recent work,^35^ using the experimentally measured temperature dependence of water dielectric constant given in ref (42), we showed that at room temperature, the change of water reorganization entropy −*T*Δ*S*_el_ and energy (enthalpy) Δ*H*_el_ in electrostatic interaction calculated by eqs 1 and 2 yields, respectively,
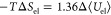
4and
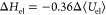
5where ⟨*U*_el_⟩ is the average of the sum of all effective ionic pair interactions given by eq 3. Equations 4 and 5 highlight the surprising result that electrostatic assembly in water at room temperature is primarily entropy-driven. The origin of this entropic driving force can be understood as arising from the increased orientational freedom of water molecules upon pairing of two oppositely charged ions,^35,47^ as illustrated in Figure 1A. This entropic contribution due to water reorganization in electrostatic interactions has been invoked to successfully explain the predominantly entropy-driven nature in polyelectrolyte complex coacervation systems.^35^ Moreover, eqs 4 and 5 show that the entropic and enthalpic contributions to the free-energy change are of opposite signs to each other. Thus, water reorganization in electrostatic interactions exhibits compensated behavior.

In the studies of biopolymer association, screened Coulomb interaction in the form of
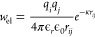
6is often used to account for the effects of salt ions, where κ is the inverse Debye screen length given by , with *e* the electronic charge, and *C* the concentration of salt (assumed to be monovalent here). In this work, we use eq 6 to model electrostatic interactions between the biopolymers with the salt concentration at 0.1 M to mimic the typical salt condition in cellular environments. In the wide temperature range of interest for this work, the Debye screening length changes little,^48^ so we take κ to be constant. As a result, eqs 4 and 5 remain valid for calculating the entropic and enthalpic contributions from water reorganization in biomolecular association with screened electrostatics.

### Water Reorganization in Temperature-Dependent Hydrophobic Interactions

Hydrophobic interaction refers to the effective non-ionic interaction between solutes that results from the change of local water structure and the adsorption/release of water molecules.^44,49^ These water reorganization events involve both entropy and enthalpy changes.^38^ As illustrated in Figure 1B, depending on whether water molecules tend to push two solutes together or away, the effective hydrophobic interaction can be attractive or repulsive (here we consider hydrophilic interaction as a special case of the hydrophobic interaction where the solutes repel each other).

Hydrophobic interactions are known to be strongly temperature-dependent.^38,43,44,49^ The temperature-dependent hydrophobic interactions greatly influence the behavior of biomolecules, such as protein folding^43,50^ and condensation.^36,51^ Effective, temperature-dependent hydrophobic interaction potentials have been recently developed to model the LCST and UCST liquid–liquid phase separation of disordered proteins.^36,52^ Following a similar strategy to that in ref (36), we construct a generic temperature-dependent hydrophobic potential that allows the water reorganization entropy and energy to be easily extracted using eqs 1 and 2. Our model contains a modified Weeks–Chandler–Andersen (WCA) potential, *u*_wca(50,49)_, to capture the short-range hard-core repulsion^53^ and a temperature-dependent term *u*_T_ to describe the effect of solvent quality:
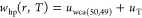
7The generalized WCA potential has the form
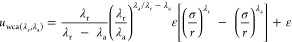
8with a cutoff , where λ_r_ = 50 and λ_a_ = 49. ε = *k*_B_*T*. *u*_wca(50,49)_ is a more faithful representation of the hard-sphere repulsion than the more traditional *u*_wca(12,6)_,^53^ as shown in Figure S1, Supporting Information. Below, we will write *u*_wca(50,49)_ as *u*_wca_ for simplicity. As an interaction mimicking the hard-sphere repulsion, *u*_wca_ is primarily entropic in origin, and for this reason, we choose ε = *k*_B_*T* in our simulation. However, as shown in Figure S2, Supporting Information, the change in the interaction due to this potential in the binding between the two polymers studied in this work is very small, so the precise choice of ε is inconsequential.

For the temperature-dependent term, we follow a commonly assumed form^36,54,55^
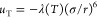
9where the coefficient λ(*T*) captures the different solvent conditions for the solute. At the level of the second virial coefficient *B*_2_, the solvent condition is characterized by the θ temperature, at which *B*_2_ = 0. For this reason, we write the coefficient λ(*T*) as
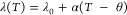
10where α describes the sensitivity of the temperature. In principle, it is possible to include higher-order terms in *T* – θ for quantitative accuracy. Here, we use the linear form for convenience and simplicity; for the temperature range of interest in this work, the linear form is sufficient to capture the temperature response of the hydrophobic interaction. λ_0_ is a constant, whose value is determined by the vanishing of *B*_2_ at *T* = θ. From the expression for *B*_2_
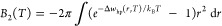
11we determine λ_0_ ≈ 0.95. The sign of α in eq 10 distinguishes between LCST and UCST behavior: If α > 0, increasing temperature results in a stronger effective solute–solute attraction that can eventually lead to phase separation, corresponding to LCST; if α < 0, the attraction is enhanced by decreasing the temperature, corresponding to UCST.

We comment that the concepts of the θ temperature, UCST, and LCST, are usually employed to describe interactions between the same solute molecules. However, the general form of λ(*T*) can be used to describe cross-interactions between different solute molecules. In that case, θ refers to the temperature at which the second virial coefficient between two different molecular species vanishes.

Experimental determination of the θ temperature for the different moieties in the biomolecular association is challenging, and such data are at present not available. However, in most experiments concerning the temperature-dependent biomolecular association, structural changes or phase transitions typically take place in the range of (−100, 100)K from room temperature.^50,51,56,57^ Thus, in this study, we consider (*T* – θ) in the range of (−100, 100)K to represent the most experimentally relevant conditions.

**Figure 2:**
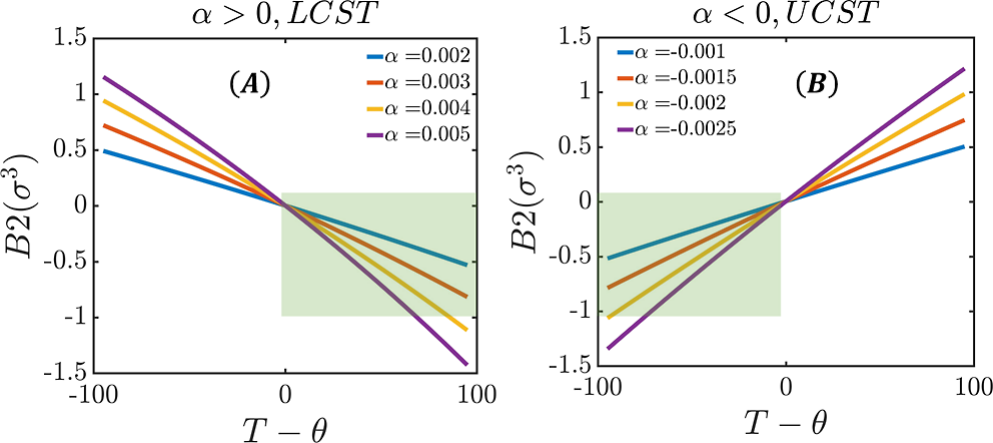
Second virial coefficient due to the hydrophobic interaction as a function of the difference between the room temperature and the theta temperature (*T* – θ). (A) LCST system. (B) UCST system. The shaded area in each figure shows the experimentally relevant conditions for biomolecular association.

Figure 2 shows the second virial coefficient calculated from eq 11, as a function of the temperature difference *T* – θ. For both the LCST and UCST cases, in the range of parameters that yield reasonable values for *B*_2_, its temperature dependence is approximately linear, especially close to the theta point, as expected on physical grounds.

Using eqs 1 and 10, the entropic contribution to the free energy from water reorganization in the hydrophobic interaction can be obtained as
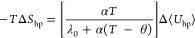
12and the corresponding enthalpic contribution calculated using eq 2 yields
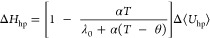
13where again ⟨*U*_hp_⟩ refers to the average of the sum of all pair interactions due to the potential *u*_T_. From eqs 12 and 13, we see that variations in the parameter α and the temperature difference *T* – θ result in variations of the entropic and enthalpic contributions to the free-energy change and that these two contributions compensate each other.

### Other Simulation Details

Our simulation uses an implicit-solvent representation, with dielectric constant ϵ_*r*_ = 78 at *T* = 300 K. The monomer size is set at σ = 6.0 Å for all monomers to represent the generic size of a protein residue in coarse-grained simulations.^36^ Neighboring monomers along the chains are subjected to the harmonic bond potential given by , with *K*_bond_ = 100*k*_B_*T*/σ^2^ and *r*_0_ = 0.7σ. With *k*_B_*T* being the energy scale in the bond potential, the bonding interaction should be considered as entropic in nature. Using eqs 1 and 2, we get
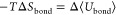
14and
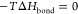
15However, similar to *U*_wca_, the change in *U*_bond_ contributes minimally to the PMF (see Figure S2, Supporting Information); thus, the precise designation of *U*_wca_ and *U*_bond_ interaction energies is immaterial.

The total entropy of complexation is calculated by the PMF and all known enthalpy contributions
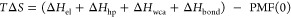
16where PMF(0) is the free energy of complexation.

The conformation entropy is then computed by subtracting all known entropy (that can be calculated directly from the interaction potentials) from the total entropy
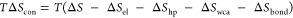
17Note that the translational entropy of the polymers is not considered here.

All simulations are performed at *T* = 300 K with a Langevin thermostat using the large-scale atomic/molecular massively parallel simulator (LAMMPS) platform. The simulation time scale is given by  where the mass of the monomer *m* is set at 1. The positions and velocities of the beads are updated with an integration time step Δ*t* = 0.002τ. The simulation box has dimensions 100σ × 100σ × 100σ. Each polymer pair is equilibrated for 10^6^τ before performing the PMF calculations.

We calculate the PMF of association using the adaptive bias force algorithm^58,59^ implemented in LAMMPS.^60^ The coordinate of the PMF is taken to be the center-of-mass distance between the two chains. We sample the distance *r* in the range 0–30σ. The distance range is divided into 3 consecutive windows of 0–3σ, 3–10σ, and 10–30σ to improve the efficiency of the PMF calculations.^59^ Each window is further divided into bins with equal widths of 0.5σ. For each window, we perform the simulation for 1 × 10^7^τ to reach convergence. Other details in the PMF calculation are the same as in our previous work.^35^

## Results and Discussion

### Free Energy and Pathway of Association between Two Oppositely Charged Polymers

**Figure 3:**
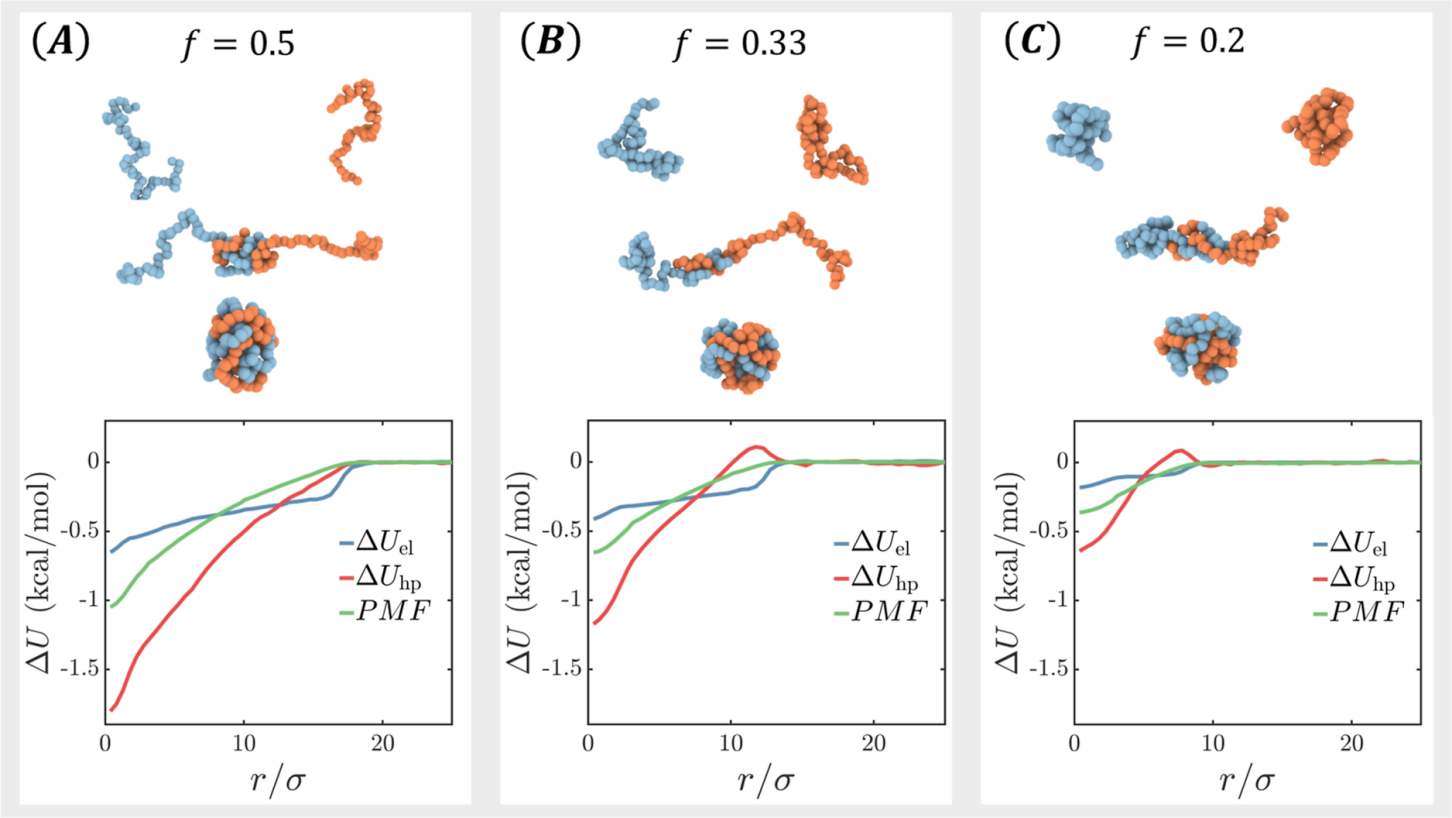
PMF and the corresponding changes in electrostatic and hydrophobic interactions for the association of two oppositely charged chains at different charge fractions *f*. (A) *f* = 0.5: the two chains are extended. (B) *f* = 0.3: the two chains are coil-like. (C) *f* = 0.1: the two chains are globule-like. All monomers interact with the same hydrophobic attraction corresponding to *B*_2_ = −1σ^3^.

To illustrate our approach, we simulate the association between two generic polyelectrolyte chains as a crude model for charged biomacromolecules. The two model polymers each have 60 beads connected by harmonic bonds. The two chains are oppositely charged with the same charge fraction *f*, which is controlled by placing unit charges regularly spaced along the chain backbone. All beads have the same hydrophobic interaction with the same second virial coefficient *B*_2_. The conformation of the chains before association depends on the charge fraction *f* and *B*_2_. For *B*_2_ = −σ^3^, by tuning *f*, we can model a chain that takes extended (Figure 3A), coil-like (Figure 3B), and globule-like (Figure 3C) conformations, mimicking intrinsically disordered proteins to folded proteins. Below, for simplicity and to connect with the literature on biomolecular systems, we call a chain in the extended or coil-like state a disordered chain and a chain in the globule-like state a folded chain.

The bottom row of Figure 3 shows the free energy (PMF), *U*_hp_, and *U*_el_ upon complexation between the two chains. On the per residue basis, the binding free energy is around −0.5 to – 1.0 kcal/mol, similar to the experimental values for protein–protein^9^ and protein–ligand^20^ association. The complexation between the disordered chains is stronger than between the folded chains. Despite *B*_2_ being the same in all three cases, *U*_hp_ decreases more in the case of higher charge fraction due to the favorable hydrophobic contacts brought about by the electrostatic attractions between the two chains.

Interestingly, the complexation pathway between two folded chains shows that *U*_hp_ first increases when the two chains start to contact and then decreases as they fuse together. This increase in *U*_hp_ is a result of temporary unfolding of the chains as they are drawn together by the electrostatic attraction; see the snapshots in Figure 3B,C.

With the PMF, *U*_hp_, and *U*_el_ calculated this way, we are in a position to address the issue of EEC by varying the parameters in the model, as detailed below.

### Enthalpy–Entropy Compensation Due to Water Reorganization

To study the EEC, we perform extensive PMF calculations for the complexation between two oppositely charged polymers by varying α and *T* – θ in the hydrophobic interaction and the charge fraction *f*. To ensure the physical relevance of our simulations, we consider the range of *T* – θ to be (−100, 100)K, and we constrain the second virial coefficient due to the hydrophobic interaction to be within (−1.1, 0.1)σ^3^. Within the limits for *B*_2_ and *T* – θ, we vary α in the range of |α| < 0.01. To cover the different single-chain conformations, we choose three charge fractions, *f* = 0.5, 0.3, and 0.1. All simulations are performed at room temperature; therefore, by varying *T* – θ, we essentially vary the θ temperature.

**Figure 4:**
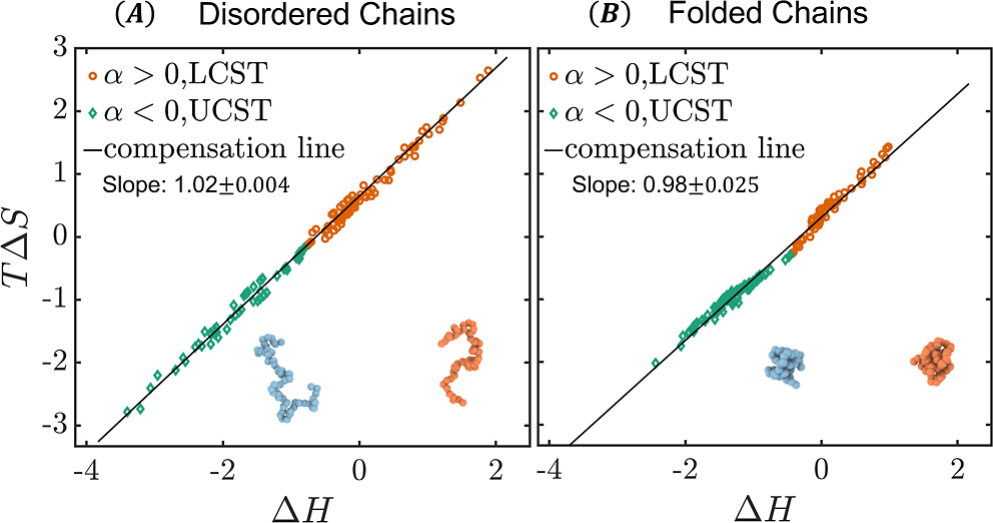
EEC in the association between (A) disordered chains and (B) folded chains. The disordered chains mostly have a charge fraction of 0.5 with some having a charge fraction of 0.3, while the folded chains mostly have a charge fraction of 0.1 with some having a charge fraction of 0.3.

**Figure 5:**
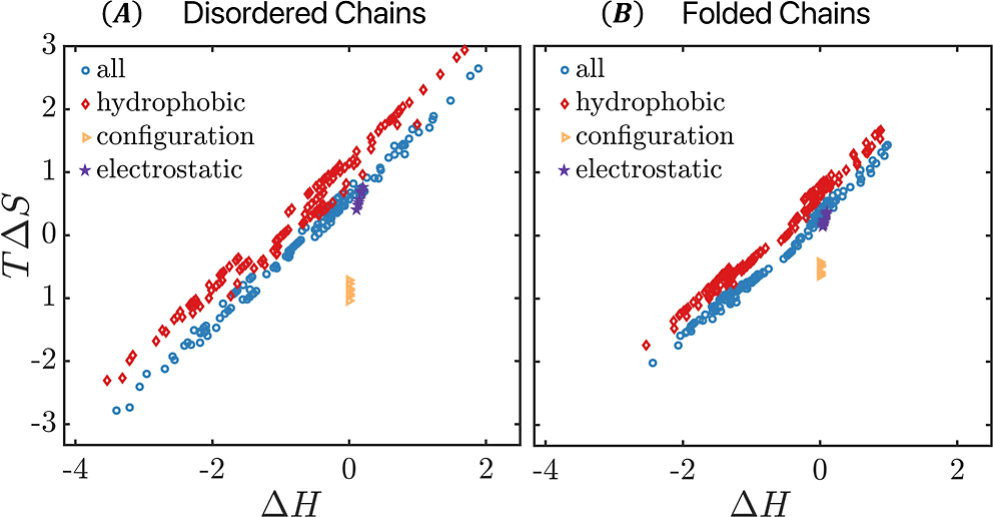
Free-energy components from hydrophobic interaction, electrostatic interaction, and configurational entropy in the association of (A) disordered chains and (B) folded chains.

Figure 4A shows the total entropy and enthalpy changes in the associations between two disordered chains, with 150 combinations of randomly chosen α and (*T* – θ). In Figure 4A, the enthalpy and entropy show clear EEC behavior; on a per residue basis, while the variation in Δ*G* is within 0.28 kcal/mol (out of ∼1.5 kcal/mol on average), the variations in *T*Δ*S* and Δ*H* span about 6 kcal/mol, with a slope close to 1 in the *T*Δ*S*–Δ*H* plot. The significant changes in enthalpy and entropy are primarily reflections of water reorganization from hydrophobic interactions calculated from eqs 12 and 13, as will be discussed further in Figure 5. These results are very similar to the experimentally observed EEC reported in ref (20) for protein–ligand binding upon single residue substitutions in the ligand: the variations in entropy and enthalpy were also about 6 kcal/mol, significantly larger than the variation in Δ*G*. The authors of ref (20) attributed the EEC to the water reorganization, which is supported by our simulation and analysis.

The association between two folded chains also exhibits a similar EEC behavior, as shown in Figure 4B. Interestingly, while the variation in Δ*G* is also small at 0.28 kcal/mol, similar to the disordered chains, the variations in entropy and enthalpy are about 3.5 kcal/mol, less than the range for the disordered chains (6 kcal/mol). These results are consistent with experimental findings by Huang and Liu^9^ that disordered proteins show more pronounced EEC behavior than folded proteins. The stronger EEC behavior in disordered chains can be explained by the fact that unfolded chains expose more residues to water before complexation, resulting in more significant water reorganization upon binding, thus leading to larger changes in enthalpy and entropy.

The EEC data in Figure 4 are naturally separated by the LCST (α > 0) and UCST (α < 0) subgroups. In the LCST association, the favorable free-energy change is dominated by solvent reorganization entropy in hydrophobic interactions, at the expense of energy. In contrast, for the UCST association, the energetic contribution from solvent reorganization dominates the free-energy change, at the cost of entropy decrease.

To get further insight into the different contributions to the enthalpy and entropy changes, we show the separate free-energy components from hydrophobic interaction, electrostatic interaction, and configuration change in Figure 5. Clearly, water reorganization is the primary cause for EEC, which is manifested through the large variations in the entropy and enthalpy changes due to the temperature-dependent hydrophobic interaction *U*_hp_. Electrostatic interaction shows only a modest compensation behavior, with the changes in *T*Δ*S*_el_ and Δ*H*_el_ significantly smaller compared to those due to the water-mediated hydrophobic interaction. There is appreciable variation in the conformation entropy change, but this variation is 1 order of magnitude smaller than the variation in the entropy change due to hydrophobic interaction.

### EEC in Biomolecular Association

Our phenomenological model for EEC behavior reported in this work is based on the interaction between a pair of homopolymers that have the same hydrophobicity on all residues. Here, EEC arises from variations in the θ point and the parameter α, which relates to LCST/ UCST. Experimentally, EEC behavior is observed in biomolecular associations involving heteropolymers such as proteins. Our analysis can be easily generalized to the association between two heteropolymers by allowing different temperature dependencies between different types of residues,^36,52^ which has been used by Mittal and co-workers to successfully describe the temperature-dependent liquid–liquid phase separation of disordered proteins.^36^ Once so generalized, the interaction between the different residues will involve different values for θ and α. EEC is then manifested through the natural variability in the value of these parameters upon residue substitutions.

We note that since the systems in our study are relatively small, it is in principle possible to compute the PMF using explicit water models. However, to address the EEC in biomolecular association, we will have to perform hundreds of such calculations using different monomer structures, which is beyond the reach of current computational resources. More importantly, we are not aware of any simple and unambiguous methods for separating the PMF into various components (electrostatic, chain conformation, and water reorganizations). Our approach provides a general and inexpensive way to extract water entropy contributions by exploiting the temperature-dependent implicit-solvent interaction potentials. Furthermore, our method can be easily extended to larger and more complex systems.

## Conclusions

By exploiting the temperature dependence in the implicit-solvent, coarse-grained interaction potentials, we are able to extract the enthalpic and entropic contributions in the free-energy change for polymer association in water. We find that under experimentally relevant conditions, water reorganization constitutes a major source of the significant variations in the entropy and enthalpy of association, which is manifested in the different temperature dependence of the hydrophobic attractions. Consequently, water reorganization results in pronounced EEC behavior: for the LCST association, water reorganization entropy dominates the free-energy change at the expense of enthalpy; for the UCST association, water reorganization enthalpy dominates the process at the expense of entropy. Other effects, such as electrostatic interaction and conformation change of the polymers, contribute much less to the variations in entropy/enthalpy.

Besides highlighting the role of water reorganization in EEC, the approach proposed in this work forms a natural coarse-grained modeling framework for understanding the thermodynamics of liquid–liquid phase separation involving polymers in aqueous solutions. For example, if complex coacervation is primarily driven by electrostatic interaction, the free-energy change is dominated by the entropy gain from water reorganization, and the phase diagram should only show LCST behavior due to the decrease of the water dielectric constant with temperature. However, depending on the chemical structure of the polymers, polyelectrolyte complex coacervation can also be energy-driven or have UCST behavior.^61−64^ To fully understand the temperature dependence in the phase behavior and the thermodynamic driving forces in polyelectrolyte coacervation systems, it is necessary to include the temperature-dependent hydrophobic interaction. Furthermore, the water reorganization entropy and enthalpy highlighted in our work can be exploited for designing stable macromolecular complexes. For example, by including both LCST and UCST binding domains with a close-loop phase diagram (with LCST < UCST), the resulting macromolecular complex is expected to exhibit enhanced thermal stability with respect to a wide range of temperature variations.

## Supporting Information Available

Short-range repulsion presented by modified WCA potential and different contributions to the interaction between two polymers (PDF)

## References

[ref1] Lumry R.; Rajender S. Enthalpy-entropy compensation phenomena in water solutions of proteins and small molecules: A ubiquitous properly of water. Biopolymers 1970, 9, 1125–1227. 10.1002/bip.1970.360091002. 4918636

[ref2] Dunitz J. D. Win some, lose some: enthalpy-entropy compensation in weak intermolecular interactions. Chem. Biol. 1995, 2, 709–712. 10.1016/1074-5521(95)90097-7. 9383477

[ref3] Liu L.; Guo Q.-X. Isokinetic Relationship, Isoequilibrium Relationship, and Enthalpy-Entropy Compensation. Chem. Rev. 2001, 101, 673–696. 10.1021/cr990416z. 11712500

[ref4] Fox J. M.; Zhao M.; Fink M. J.; Kang K.; Whitesides G. M. The Molecular Origin of Enthalpy/Entropy Compensation in Biomolecular Recognition. Annu. Rev. Biophys. 2018, 47, 223–250. 10.1146/annurev-biophys-070816-033743. 29505727

[ref5] Gilli P.; Ferretti V.; Gilli G.; Borea P. A. Enthalpy-entropy compensation in drug-receptor binding. J. Phys. Chem. 1994, 98, 1515–1518. 10.1021/j100056a024.

[ref6] Olsson T. S. G.; Ladbury J. E.; Pitt W. R.; Williams M. A. Extent of enthalpy-entropy compensation in protein-ligand interactions. Protein Sci. 2011, 20, 1607–1618. 10.1002/pro.692. 21739503 PMC3190155

[ref7] Wallerstein J.; Ekberg V.; Ignjatović M. M.; Kumar R.; Caldararu O.; Peterson K.; Wernersson S.; Brath U.; Leffler H.; Oksanen E.; et al. Entropy–Entropy Compensation between the Protein, Ligand, and Solvent Degrees of Freedom Fine-Tunes Affinity in Ligand Binding to Galectin-3C. JACS Au 2021, 1, 484–500. 10.1021/jacsau.0c00094. 34467311 PMC8395690

[ref8] Reichmann D.; Rahat O.; Albeck S.; Meged R.; Dym O.; Schreiber G. The modular architecture of protein–protein binding interfaces. Proc. Natl. Acad. Sci. U.S.A. 2005, 102, 57–62. 10.1073/pnas.0407280102. 15618400 PMC544062

[ref9] Huang Y.; Liu Z. Do Intrinsically Disordered Proteins Possess High Specificity in Protein-Protein Interactions?. Chem.—Eur. J. 2013, 19, 4462–4467. 10.1002/chem.201203100. 23436397

[ref10] Bale J. B.; Gonen S.; Liu Y.; Sheffler W.; Ellis D.; Thomas C.; Cascio D.; Yeates T. O.; Gonen T.; King N. P.; et al. Accurate design of megadalton-scale two-component icosahedral protein complexes. Science 2016, 353, 389–394. 10.1126/science.aaf8818. 27463675 PMC5485857

[ref11] Jen-Jacobson L.; Engler L. E.; Jacobson L. A. Structural and Thermodynamic Strategies for Site-Specific DNA Binding Proteins. Structure 2000, 8, 1015–1023. 10.1016/s0969-2126(00)00501-3. 11080623

[ref12] Privalov P. L.; Dragan A. I.; Crane-Robinson C.; Breslauer K. J.; Remeta D. P.; Minetti C. A. What Drives Proteins into the Major or Minor Grooves of DNA?. J. Mol. Biol. 2007, 365, 1–9. 10.1016/j.jmb.2006.09.059. 17055530 PMC1934558

[ref13] Tzeng S.-R.; Kalodimos C. G. Protein activity regulation by conformational entropy. Nature 2012, 488, 236–240. 10.1038/nature11271. 22801505

[ref14] Nott T.; Petsalaki E.; Farber P.; Jervis D.; Fussner E.; Plochowietz A.; Craggs T. D.; Bazett-Jones D.; Pawson T.; Forman-Kay J.; et al. Phase Transition of a Disordered Nuage Protein Generates Environmentally Responsive Membraneless Organelles. Mol. Cell 2015, 57, 936–947. 10.1016/j.molcel.2015.01.013. 25747659 PMC4352761

[ref15] Wei M.-T.; Elbaum-Garfinkle S.; Holehouse A. S.; Chen C. C.-H.; Feric M.; Arnold C. B.; Priestley R. D.; Pappu R. V.; Brangwynne C. P. Phase behaviour of disordered proteins underlying low density and high permeability of liquid organelles. Nat. Chem. 2017, 9, 1118–1125. 10.1038/nchem.2803. 29064502 PMC9719604

[ref16] Brady J. P.; Farber P. J.; Sekhar A.; Lin Y.-H.; Huang R.; Bah A.; Nott T. J.; Chan H. S.; Baldwin A. J.; Forman-Kay J. D.; et al. Structural and hydrodynamic properties of an intrinsically disordered region of a germ cell-specific protein on phase separation. Proc. Natl. Acad. Sci. U.S.A. 2017, 114, E8194–E8203. 10.1073/pnas.1706197114. 28894006 PMC5625912

[ref17] Gao A.; Shrinivas K.; Lepeudry P.; Suzuki H. I.; Sharp P. A.; Chakraborty A. K. Evolution of weak cooperative interactions for biological specificity. Proc. Natl. Acad. Sci. U.S.A. 2018, 115, E11053–E11060. 10.1073/pnas.1815912115. 30404915 PMC6255166

[ref18] Searle M. S.; Williams D. H. The cost of conformational order: entropy changes in molecular associations. J. Am. Chem. Soc. 1992, 114, 10690–10697. 10.1021/ja00053a002.

[ref19] Ahmad M.; Helms V.; Lengauer T.; Kalinina O. V. Enthalpy–Entropy Compensation upon Molecular Conformational Changes. J. Chem. Theory Comput. 2015, 11, 1410–1418. 10.1021/ct501161t. 26574352

[ref20] Breiten B.; Lockett M. R.; Sherman W.; Fujita S.; Al-Sayah M.; Lange H.; Bowers C. M.; Heroux A.; Krilov G.; Whitesides G. M. Water Networks Contribute to Enthalpy/Entropy Compensation in Protein–Ligand Binding. J. Am. Chem. Soc. 2013, 135, 15579–15584. 10.1021/ja4075776. 24044696

[ref21] Dragan A. I.; Read C. M.; Crane-Robinson C. Enthalpy–entropy compensation: the role of solvation. Eur. Biophys. J. 2017, 46, 301–308. 10.1007/s00249-016-1182-6. 27796417 PMC5384952

[ref22] Grunwald E.; Steel C. Solvent reorganization and thermodynamic enthalpy-entropy compensation. J. Am. Chem. Soc. 1995, 117, 5687–5692. 10.1021/ja00126a009.

[ref23] Starikov E. Valid entropy–enthalpy compensation: Fine mechanisms at microscopic level. Chem. Phys. Lett. 2013, 564, 88–92. 10.1016/j.cplett.2013.02.016.

[ref24] Tanford C. The Hydrophobic Effect and the Organization of Living Matter. Science 1978, 200, 1012–1018. 10.1126/science.653353. 653353

[ref25] Chaplin M. Do we underestimate the importance of water in cell biology?. Nat. Rev. Mol. Cell Biol. 2006, 7, 861–866. 10.1038/nrm2021. 16955076

[ref26] Levy Y.; Onuchic J. N. Water mediation in protein folding and molecular recognition. Annu. Rev. Biophys. Biomol. Struct. 2006, 35, 389–415. 10.1146/annurev.biophys.35.040405.102134. 16689642

[ref27] Rego N. B.; Xi E.; Patel A. J. Protein Hydration Waters Are Susceptible to Unfavorable Perturbations. J. Am. Chem. Soc. 2019, 141, 2080–2086. 10.1021/jacs.8b11448. 30615413

[ref28] Rego N. B.; Xi E.; Patel A. J. Identifying hydrophobic protein patches to inform protein interaction interfaces. Proc. Natl. Acad. Sci. U.S.A. 2021, 118, 118 10.1073/pnas.2018234118. PMC8018078 33526682

[ref29] Ahlers J.; Adams E. M.; Bader V.; Pezzotti S.; Winklhofer K. F.; Tatzelt J.; Havenith M. The key role of solvent in condensation: Mapping water in liquid-liquid phase-separated FUS. Biophys. J. 2021, 120, 1266–1275. 10.1016/j.bpj.2021.01.019. 33515602 PMC8059208

[ref30] Clarke S. The hydrophobic effect: Formation of micelles and biological membranes, 2nd edition (Tanford, Charles). J. Chem. Educ. 1981, 58, A246 10.1021/ed058pa246.1.

[ref31] Maibaum L.; Dinner A. R.; Chandler D. Micelle Formation and the Hydrophobic Effect. J. Phys. Chem. B 2004, 108, 6778–6781. 10.1021/jp037487t.

[ref32] Abel R.; Young T.; Farid R.; Berne B. J.; Friesner R. A. Role of the Active-Site Solvent in the Thermodynamics of Factor Xa Ligand Binding. J. Am. Chem. Soc. 2008, 130, 2817–2831. 10.1021/ja0771033. 18266362 PMC2761766

[ref33] Biela A.; Nasief N. N.; Betz M.; Heine A.; Hangauer D.; Klebe G. Dissecting the Hydrophobic Effect on the Molecular Level: The Role of Water, Enthalpy, and Entropy in Ligand Binding to Thermolysin. Angew. Chem., Int. Ed. 2013, 52, 1822–1828. 10.1002/anie.201208561. 23283700

[ref34] Portman K. L.; Long J.; Carr S.; Briand L.; Winzor D. J.; Searle M. S.; Scott D. J. Enthalpy/Entropy Compensation Effects from Cavity Desolvation Underpin Broad Ligand Binding Selectivity for Rat Odorant Binding Protein 3. Biochemistry 2014, 53, 2371–2379. 10.1021/bi5002344. 24665925

[ref35] Chen S.; Wang Z.-G. Driving force and pathway in polyelectrolyte complex coacervation. Proc. Natl. Acad. Sci. U.S.A. 2022, 119, e2209975119 10.1073/pnas.2209975119. 36037377 PMC9457374

[ref36] Dignon G. L.; Zheng W.; Kim Y. C.; Mittal J. Temperature-Controlled Liquid–Liquid Phase Separation of Disordered Proteins. ACS Cent. Sci. 2019, 5, 821–830. 10.1021/acscentsci.9b00102. 31139718 PMC6535772

[ref37] Wu J.; Prausnitz J. M. Pairwise-additive hydrophobic effect for alkanes in water. Proc. Natl. Acad. Sci. U.S.A. 2008, 105, 9512–9515. 10.1073/pnas.0802162105. 18599448 PMC2474496

[ref38] Ben-Amotz D. Water-Mediated Hydrophobic Interactions. Annu. Rev. Phys. Chem. 2016, 67, 617–638. 10.1146/annurev-physchem-040215-112412. 27215821

[ref39] Jolicoeur C.; Philip P. R. Enthalpy–Entropy Compensation for Micellization and Other Hydrophobic Interactions in Aqueous Solutions. Can. J. Chem. 1974, 52, 1834–1839. 10.1139/v74-262.

[ref40] Evans D. F.; Ninham B. W. Ion binding and the hydrophobic effect. J. Phys. Chem. 1983, 87, 5025–5032. 10.1021/j150642a050.

[ref41] Zangi R.; Hagen M.; Berne B. J. Effect of Ions on the Hydrophobic Interaction between Two Plates. J. Am. Chem. Soc. 2007, 129, 4678–4686. 10.1021/ja068305m. 17378564

[ref42] Malmberg C. G.; Maryott A. A. Dielectric constant of water from 0 to 100 C. J. Res. Natl. Bur. Stand. 1956, 56, 1–8. 10.6028/jres.056.001.

[ref43] Baldwin R. L. Temperature dependence of the hydrophobic interaction in protein folding. Proc. Natl. Acad. Sci. U.S.A. 1986, 83, 8069–8072. 10.1073/pnas.83.21.8069. 3464944 PMC386868

[ref44] Meyer E. E.; Rosenberg K. J.; Israelachvili J. Recent progress in understanding hydrophobic interactions. Proc. Natl. Acad. Sci. U.S.A. 2006, 103, 15739–15746. 10.1073/pnas.0606422103. 17023540 PMC1635073

[ref45] Fröhlich H. Theory of Dielectrics: Dielectric Constant and Dielectric Loss; Clarendon Press: Oxford, 1958.

[ref46] Muthukumar M. Physics of Charged Macromolecules; Cambridge University Press, 2023.

[ref47] Varner S.; Balzer C.; Wang Z.-G. Entropic Origin of Ionic Interactions in Polar Solvents. J. Phys. Chem. B 2023, 127, 4328–4337. 10.1021/acs.jpcb.3c00588. 37159929 PMC10201535

[ref48] Jacob I. Intermolecular and surface forces; Academic Press, 2011.

[ref49] Southall N. T.; Dill K. A.; Haymet A. D. J. A View of the Hydrophobic Effect. J. Phys. Chem. B 2002, 106, 2812–3533. 10.1021/jp020104r.

[ref50] van Dijk E.; Hoogeveen A.; Abeln S. The Hydrophobic Temperature Dependence of Amino Acids Directly Calculated from Protein Structures. PLoS Comput. Biol. 2015, 11, e1004277 10.1371/journal.pcbi.1004277. 26000449 PMC4441443

[ref51] Quiroz F. G.; Chilkoti A. Sequence heuristics to encode phase behaviour in intrinsically disordered protein polymers. Nat. Mater. 2015, 14, 1164–1171. 10.1038/nmat4418. 26390327 PMC4618764

[ref52] Baul U.; Bley M.; Dzubiella J. Thermal Compaction of Disordered and Elastin-like Polypeptides: A Temperature-Dependent, Sequence-Specific Coarse-Grained Simulation Model. Biomacromolecules 2020, 21, 3523–3538. 10.1021/acs.biomac.0c00546. 32692541

[ref53] Jover J.; Haslam A. J.; Galindo A.; Jackson G.; Müller E. A. Pseudo hard-sphere potential for use in continuous molecular-dynamics simulation of spherical and chain molecules. J. Chem. Phys. 2012, 137, 144505 10.1063/1.4754275. 23061853

[ref54] Maerzke K. A.; Siepmann J. I. Transferable Potentials for Phase Equilibria-Coarse-Grain Description for Linear Alkanes. J. Phys. Chem. B 2011, 115, 3452–3465. 10.1021/jp1063935. 21395331

[ref55] Griffiths M. Z.; Shinoda W. tSPICA: Temperature- and Pressure-Dependent Coarse-Grained Force Field for Organic Molecules. J. Chem. Inf. Model. 2019, 59, 3829–3838. 10.1021/acs.jcim.9b00480. 31398283

[ref56] Lüdemann S.; Abseher R.; Schreiber H.; Steinhauser O. The Temperature-Dependence of Hydrophobic Association in Water. Pair versus Bulk Hydrophobic Interactions. J. Am. Chem. Soc. 1997, 119, 4206–4213. 10.1021/ja953439d.

[ref57] Chen W.-Y.; Huang H.-M.; Lin C.-C.; Lin F.-Y.; Chan Y.-C. Effect of Temperature on Hydrophobic Interaction between Proteins and Hydrophobic Adsorbents: Studies by Isothermal Titration Calorimetry and the van’t Hoff Equation. Langmuir 2003, 19, 9395–9403. 10.1021/la034783o.

[ref58] Darve E.; Rodríguez-Gómez D.; Pohorille A. Adaptive biasing force method for scalar and vector free energy calculations. J. Chem. Phys. 2008, 128, 144120 10.1063/1.2829861. 18412436

[ref59] Comer J.; Gumbart J. C.; Hénin J.; Lelièvre T.; Pohorille A.; Chipot C. The Adaptive Biasing Force Method: Everything You Always Wanted To Know but Were Afraid To Ask. J. Phys. Chem. B 2015, 119, 1129–1151. 10.1021/jp506633n. 25247823 PMC4306294

[ref60] Fiorin G.; Klein M. L.; Hénin J. Using collective variables to drive molecular dynamics simulations. Mol. Phys. 2013, 111, 3345–3362. 10.1080/00268976.2013.813594.

[ref61] Kim H.; Jeon B.-j.; Kim S.; Jho Y.; Hwang D. S. Upper Critical Solution Temperature (UCST) Behavior of Coacervate of Cationic Protamine and Multivalent Anions. Polymers 2019, 11, 691 10.3390/polym11040691. 30995741 PMC6523134

[ref62] Ye Z.; Sun S.; Wu P. Distinct Cation–Anion Interactions in the UCST and LCST Behavior of Polyelectrolyte Complex Aqueous Solutions. ACS Macro Lett. 2020, 9, 974–979. 10.1021/acsmacrolett.0c00303. 35648610

[ref63] Girard M.; Turgeon S. L.; Gauthier S. F. Thermodynamic Parameters of β-Lactoglobulin-Pectin Complexes Assessed by Isothermal Titration Calorimetry. J. Agric. Food Chem. 2003, 51, 4450–4455. 10.1021/jf0259359. 12848524

[ref64] Harnsilawat T.; Pongsawatmanit R.; McClements D. Characterization of β-lactoglobulin–sodium alginate interactions in aqueous solutions: A calorimetry, light scattering, electrophoretic mobility and solubility study. Food Hydrocolloids 2006, 20, 577–585. 10.1016/j.foodhyd.2005.05.005.

