# Supplementary material for: Using Implicit-Solvent Potentials to Extract Water Contributions to Enthalpy–Entropy Compensation in Biomolecular Associations
Source: J Phys Chem B. 2023 Jul 26;127(30):6825–32. doi: 10.1021/acs.jpcb.3c03799 (PMC10405215; doi:10.1021/acs.jpcb.3c03799)
Supplement: Supplementary file 1 — jp3c03799_si_001.pdf [file jp3c03799_si_001.pdf]

Supporting Information: Using Implicit-solvent Potentials to Extract Water Contributions to Enthalpy–Entropy Compensation in Biomolecular Association

Shensheng Chen and Zhen-Gang Wang*

Division of Chemistry and Chemical Engineering,1200 E California Blvd,California Institute of Technology, Pasadena, California 91125, United States

E-mail: zgw@caltech.edu

Short-range repulsion represented by modified WCA potential

Figure S1 shows the difference between $U_{\text{wca}(50,49)}$ and $U_{\text{wca}(12,6)}$ on representing the short-range repulsion. Clearly, $U_{\text{wca}(50,49)}$ is a better representation of the hard-core repulsion (an entropy effect), as its dominance in the short distances is affected less by the attraction term.

Different contributions to the interaction between two polymers

Figure S2 shows all the different contributions to the interaction between two disordered chains (left) and two folded chains (right), corrsponding to the main text in Fig. 3A and Fig. 3C. Changes in U_{wca} and U_{bond} are very small compared to other components.

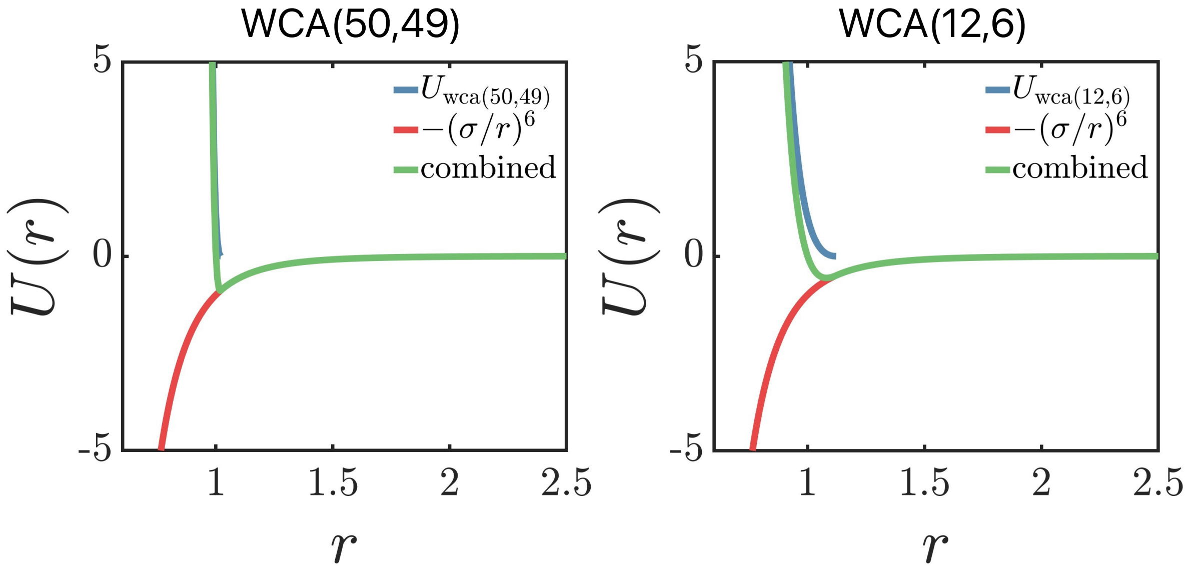

Figure S1: Difference between $U_{\mathrm{wca}(50,49)}$ (blue curve, left) and $U_{\mathrm{wca}(12,6)}$ (blue curve, right) in short-range repulsion.

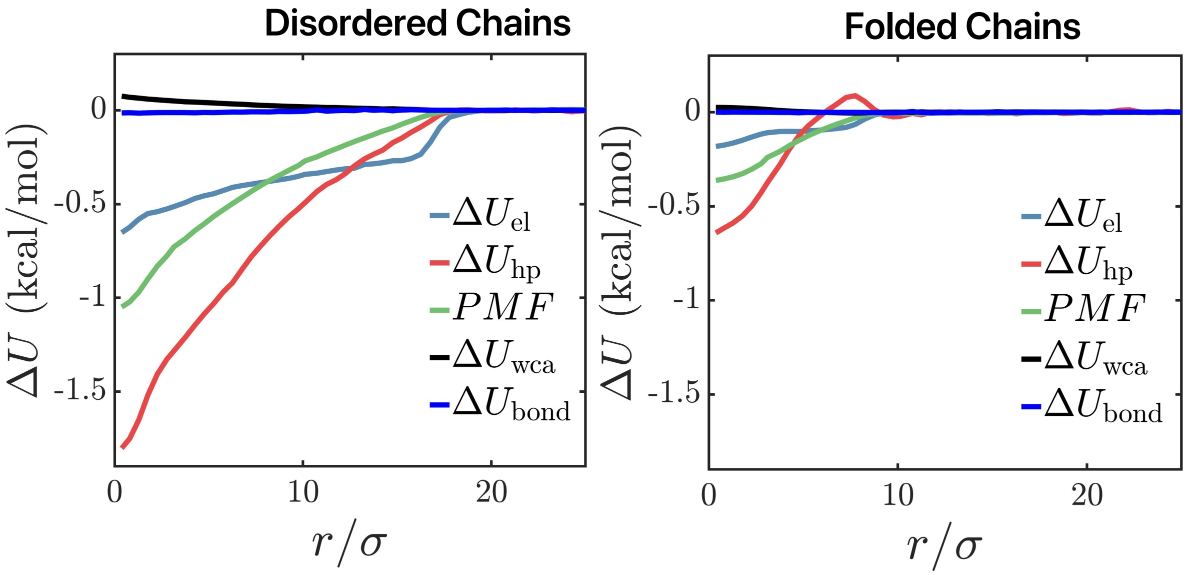

Figure S2: Different contributions to the interaction between two disordered chains (left), and between two folded chains (right).
